# Resveratrol Treatment Reduces Cardiac Progenitor Cell Dysfunction and Prevents Morpho-Functional Ventricular Remodeling in Type-1 Diabetic Rats

**DOI:** 10.1371/journal.pone.0039836

**Published:** 2012-06-29

**Authors:** Francesca Delucchi, Roberta Berni, Caterina Frati, Stefano Cavalli, Gallia Graiani, Roberto Sala, Christine Chaponnier, Giulio Gabbiani, Luca Calani, Daniele Del Rio, Leonardo Bocchi, Costanza Lagrasta, Federico Quaini, Donatella Stilli

**Affiliations:** 1 Dipartimento Biologia Evolutiva e Funzionale, Università di Parma, Parma, Italy; 2 Dipartimento di Patologia e Medicina di Laboratorio, Università di Parma, Parma, Italy; 3 Dipartimento di Medicina Sperimentale, Università di Parma, Parma, Italy; 4 Department of Pathology and Immunology, University of Geneva, Geneva, Switzerland; 5 Dipartimento di Sanità Pubblica, Università di Parma, Parma, Italy; 6 Dipartimento di Medicina Interna e Scienze Biomediche, Università di Parma, Parma, Italy; 7 Centro Interdipartimentale Cellule Staminali Cardiache, Università di Parma, Parma, Italy; University of Michigan Medical School, United States of America

## Abstract

Emerging evidence suggests that both adult cardiac cell and the cardiac stem/progenitor cell (CSPC) compartments are involved in the patho-physiology of diabetic cardiomyopathy (DCM). We evaluated whether early administration of Resveratrol, a natural antioxidant polyphenolic compound, in addition to improving cardiomyocyte function, exerts a protective role on (i) the progenitor cell pool, and (ii) the myocardial environment and its impact on CSPCs, positively interfering with the onset of DCM phenotype. Adult Wistar rats (n = 128) with streptozotocin-induced type-1 diabetes were either untreated (D group; n = 54) or subjected to administration of trans-Resveratrol (i.p. injection: 2.5 mg/Kg/day; DR group; n = 64). Twenty-five rats constituted the control group (C). After 1, 3 or 8 weeks of hyperglycemia, we evaluated cardiac hemodynamic performance, and cardiomyocyte contractile properties and intracellular calcium dynamics. Myocardial remodeling and tissue inflammation were also assessed by morphometry, immunohistochemistry and immunoblotting. Eventually, the impact of the diabetic “milieu” on CSPC turnover was analyzed in co-cultures of healthy CSPCs and cardiomyocytes isolated from D and DR diabetic hearts. In untreated animals, cardiac function was maintained during the first 3 weeks of hyperglycemia, although a definite ventricular remodeling was already present, mainly characterized by a marked loss of CSPCs and adult cardiac cells. Relevant signs of ventricular dysfunction appeared after 8 weeks of diabetes, and included: 1) a significant reduction in ±dP/dt in comparison with C group, 2) a prolongation of isovolumic contraction/relaxation times, 3) an impaired contraction of isolated cardiomyocytes associated with altered intracellular calcium dynamics. Resveratrol administration reduced atrial CSPC loss, succeeded in preserving the functional abilities of CSPCs and mature cardiac cells, improved cardiac environment by reducing inflammatory state and decreased unfavorable ventricular remodeling of the diabetic heart, leading to a marked recovery of ventricular function. These findings indicate that RSV can constitute an adjuvant therapeutic option in DCM prevention.

## Introduction

Diabetes mellitus is an independent risk factor for left ventricular dysfunction and is associated with a specific cardiomyopathy (diabetic cardiomyopathy: DCM). DCM develops independently of common co-morbidities [1]–[2] and can be considered a maladaptation of the heart mostly driven by the metabolic derangements that accompany diabetes mellitus. Hyperglycemia and dyslipidemia seem to be central in the pathogenesis of DCM via a series of maladaptive stimuli among which oxidative stress has a pivotal role [3]. Cellular oxidative stress leads to nuclear and mitochondrial DNA damage, altered gene expression, apoptotic cell death, interstitial inflammation, myocardial fibrosis and collagen accumulation [1], [4]–[6], favoring the occurrence of DCM phenotype.

Recent studies reported that high glucose levels lead to endothelial progenitor cell (EPC) senescence, and impaired proliferation, adhesion, and migration capacities [7]–[8] resulting in decreased neoangiogenesis. Emerging evidence also suggests that the cardiac stem/progenitor cell (CSPC) compartment is involved in the occurrence of diabetic cardiac dysfunction [9]. Hyperglycemia-induced CSPC damage may negatively affect cardiac structure and function playing a specific and relevant role in the pathophysiology of DCM that develops independently of vascular disease.

It has been shown that several sub-populations of CSPCs reside within the adult heart and possess the ability to differentiate into all constituent cells of cardiac tissue including cardiomyocytes, vascular smooth muscle, endothelial cells and fibroblasts [10]. In early phases of diabetes, the activation of CSPCs associated with proliferation of functionally competent and properly integrated myocytes was shown to represent a compensatory mechanism to counteract cell loss and to preserve cardiac electromechanical performance [11]. However, later phases of diabetes are characterized by progressive CSPC damage and exhaustion associated with loss of the physiologic cell turnover, leading to overt ventricular dysfunction [9]. Thus, strategies aimed at preserving both parenchymal cells and the pool of progenitor cells responsible for tissue homeostasis may represent an innovative approach for DCM prevention and treatment.

Reactive oxygen species (ROS) modulate progenitor cell growth and survival: high levels of oxidative stress trigger cellular damage pathways and are critically involved in CSPC loss [12]. It follows that therapies able to reduce ROS and to enhance ROS scavenging systems should have therapeutic efficacy in preventing CSPC damage. Resveratrol (RSV: trans-3,5,4′-trihydroxystilbene), a polyphenolic compound and naturally occurring phytoalexin present in red wine and vegetable foods, might constitute an effective therapeutic adjuvant to prevent the occurrence of DCM. Besides its antioxidant, anti-apoptotic/anti-inflammatory effects, RSV was shown to exert several other cardioprotective actions, in both ischemic and diabetic rat heart [13]–[14]. In experimental models of diabetes, it has been reported that RSV administration reduces reactive oxygen species [15] and the incidence of cardiomyocyte death [16]–[17], and improves cardiac function by enhancing the expression of sarcoplasmic calcium ATPase (SERCA2a) in cardiomyocytes, through the activation of deacetylase silent information regulator 2/sirtuin 1 (SIRT1) [18]–[19]. Recent data also indicate that RSV may exert beneficial effects on cultured human circulating endothelial progenitor cells, at least in part mediated by inhibited p38-phosphorylation and enhanced NO levels [20]. Finally, in rat models of myocardial infarction subjected to stem-cell regenerative therapy, pre-treatment with RSV was shown to enhance stem cell survival and proliferation [21].

Up to now RSV-induced improvement in the number and function of adult CSPCs in the diabetic heart remains to be explored. In the present study we addressed this issue in a rat model of type-1 diabetes. Following an approach from intact animal to tissue-cellular-molecular level, we tested the hypothesis that early administration of RSV can prevent the onset and development of DCM phenotype. We evaluated the potential protective role of RSV at different times after the induction of hyperglycemia on (i) the progenitor cell compartment, (ii) the myocardial environment and its impact on CSPC survival and functional properties, (iii) the changes in the relative expression of the skeletal actin isoform usually associated with the progression of myocardial damage [22]. The functional counterpart of the above processes was also evaluated, in terms of hemodynamics and cardiomyocyte contractile properties.

We found that early RSV administration succeeded in preserving the numerical density and functional abilities of both CSPCs and mature cardiac cells, improved cardiac environment by reducing inflammatory state and decreased unfavorable ventricular remodeling of the diabetic heart, leading to a marked recovery of ventricular function.

## Methods

The investigation was approved by the Veterinary Animal Care and Use Committee of the University of Parma-Italy and conforms to the National Ethical Guidelines of the Italian Ministry of Health (Permit number: 41/2009-B) and the Guide for the Care and Use of Laboratory Animals (National Institute of Health, Bethesda, MD,USA, revised 1996). All surgery was performed under ketamine chloride anesthesia, and all efforts were made to minimize suffering.

### Animals and Housing

The study population consisted of 143 male Wistar rats (Rattus norvegicus) aged 12–14 wk, weighing 373.9±2.7 g. Animals were kept in unisexual groups of four individuals from weaning (4 wk after birth) until the onset of the experiments, in a temperature-controlled room at 22–24°C, with the light on between 7.00 AM and 7.00 PM. The bedding of the cages consisted of wood shavings, and food and water were freely available. In 118 animals (Group D), diabetes was induced by a single intra-peritoneal injection of streptozotocin (STZ, 60 mg/kg) while the remaining 25 control rats (group C) were injected only with saline vehicle (0.9% NaCl). Glucose blood levels and body weights were measured in 2-hour-fasting animals, before STZ or vehicle injection, two days after injection, and then weekly until sacrifice. The blood glucose cut-off was fixed at 250 mg/dl, as measured two days after STZ injection. About 8% of the STZ-injected animals did not reach this threshold. These animals were excluded from the study. In no animals a reversal of diabetes was observed. D animals were either untreated (n = 54) or subjected to chronic administration of low doses of trans-Resveratrol (Sigma, Milan, Italy), by intraperitoneal injection of 2.5 mg/Kg/day (DR groups, n = 64). This dose of RSV was selected on the basis of preliminary results obtained in a pilot study, as described in the Supporting information (Files: Text S1, Figure S1 and Table S1). The treatment started immediately after the documented increase in glucose blood levels (2 days after STZ injection). RSV was dissolved in ethanol to prepare a stock solution (RSV concentration: 12.5 mg/mL) and stored in the dark at 4°C. For each animal, just before i.p. injection, an appropriate aliquot was taken from the stock solution and diluted in PBS to reach the desired concentration in a final volume of 200 µL. Functional measurements and sacrifice were performed 1 week (D1, D1R groups), 3 weeks (D3, D3R) or 8 weeks (D8, D8R) after induction of hyperglycemia.

### Functional Measurements

#### Hemodynamic study

Invasive hemodynamic data were recorded in 83 rats (12 D1, 17 D1R, 10 D3, 13 D3R, 8 D8, 11 D8R, and 12 C). Each rat was anaesthetized with ketamine chloride 40 mg/kg i.p. (Imalgene, Merial, Milano, Italy), plus medetomidine hydrochloride 0.15 mg/kg i.p. (Domitor, Pfizer Italia S.r.l., Latina, Italy).

The right carotid artery was cannulated with a microtip pressure transducer catheter (Millar SPC-320, Millar Instruments, Houston, TX, USA) connected to a recording system (Power Laboratory ML 845/4 channels, 2Biological Instruments, Besozzo, Italy) and systolic and diastolic blood pressures were determined. The catheter was then advanced into the left ventricle to measure: ) LV systolic pressure (LVSP), 2) LV end-diastolic pressure (LVEDP), 3) the peak rate of rise and decline of LV pressure (±dP/dt), taken as indexes of myocardial mechanical efficiency, 4) isovolumic contraction time (IVCT: duration of isovolumic contraction), and 5) LV relaxation time (LVRT), computed from –dP/dt to 5 mmHg above LVEDP [23] (software package CHART B4.2).

Then, the hearts of D, DR and C rats were divided in two subgroups and used for electrophoresis and immunoblot assay (n = 3 for each group) or morphometric and immunohistochemical analyses (see below).

#### Myocyte isolation and measurement of cell mechanics

The remaining 60 rats were used for cardiomyocyte and CSPC isolation. From the hearts of 8 D1, 8 D1R, 8 D3, 9 D3R, 8 D8, 6 D8R, and 13 C rats, individual ventricular myocytes were enzymatically isolated by collagenase perfusion in accordance with a procedure previously described [24]. Briefly, the rat heart was removed and rapidly perfused at 37°C by means of an aortic cannula with the following sequence of solutions: 1) a calcium-free solution for 5 min to remove the blood, 2) a low-calcium solution (0.1 mM) plus 1 mg/ml type 2 collagenase (Worthington Biochemical Corporation, Lakewood, NJ, USA), and 0.1 mg/ml type XIV protease (Sigma, Milan, Italy) for about 20 min, and 3) an enzyme-free, low-calcium solution for 5 min. Calcium-free solution contained the following (in mM): 126 NaCl, 22 dextrose, 5.0 MgCl_2_, 4.4 KCl, 20 taurine, 5 creatine, 5 Na pyruvate, 1 NaH_2_PO_4_, and 24 HEPES (pH = 7.4, adjusted with NaOH), and the solution was gassed with 100% O_2_.

The LV was then minced and shaken for 10 min. The cells were filtered through a nylon mesh and re-suspended in low-calcium solutions for 30 min. Then, cells were used for measuring sarcomere shortening and calcium transients. Smears were also made, and LV cells were stained with toluidine-blue. For each group, 500 cells were analyzed by optical microscopy to calculate cell surface area. Mechanical properties of ventricular myocytes were assessed by using the IonOptix fluorescence and contractility systems (IonOptix, Milton, MA, USA).

LV myocytes were placed in a chamber mounted on the stage of an inverted microscope (Nikon-Eclipse TE2000-U, Nikon Instruments, Florence, Italy) and superfused (1 ml/min at 37°C) with a Tyrode solution containing (in mM): 140 NaCl, 5.4 KCl, 1 MgCl_2_, 5 HEPES, 5.5 glucose, and 1 CaCl_2_ (pH 7.4, adjusted with NaOH). Only rod-shaped myocytes with clear edges and average sarcomere length ≥1.7 µm were selected for the analysis. All the selected myocytes did not show spontaneous contractions. The cells were field stimulated at a frequency of 1 Hz by rectangular depolarizing pulses (2 ms in duration, and twice diastolic threshold in intensity) by platinum electrodes placed on opposite sides of the chamber, connected to a MyoPacer Field Stimulator (IonOptix). The stimulated myocyte was displayed on a computer monitor using an IonOptix MyoCam camera. Load-free contraction of myocytes was measured with the IonOptix system, which records the sarcomere pattern to calculate the changes in the sarcomere spacing with a fast Fourier transform algorithm. Then, frequency data were converted to length. A total of 638 isolated ventricular myocytes were analyzed (125 from C hearts, 85 from D1, 80 from D3, 83 from D8, 111 from D1R, 95 from D3R and 59 from D8R) to assess cellular mechanical properties by computing the following parameters: mean diastolic sarcomere length, fraction of shortening (FS), and maximal rates of shortening and re-lengthening (±dL/dt). Steady-state contraction of myocytes was achieved before data recording.

In 30 cells of each group, Ca^2+^ transients were measured simultaneously with mechanical properties. Ca^2+^ transients were determined by epifluorescence after loading the myocytes with 10 µM fluo 3-AM (Invitrogen, Carlsbad, CA) for 30 min. Excitation length was 480 nm, with emission collected at 535 nm using a 40X oil objective. Fluo 3 signals were expressed as normalized fluorescence (f/f0: fold increase). The time course of the fluorescence signal decay was described by a single exponential equation, and the time constant (tau) was used as a measure of the rate of intracellular calcium clearing.

### CSPC Isolation and Cell Cultures

#### CSPC-isolation

CSPCs were isolated from the heart of control rats. The isolation procedure was the same as for cardiomyocytes, but ventricular fragments were maintained for a longer time in a collagenase+albumine solution at 37°C to allow mechanical tissue dissociation. The solution containing all cells was washed several times, centrifuged at 300 rpm to remove cardiomyocytes, and then submitted to Percoll (Sigma) gradient to further enrich the fraction of small cells. The cell layer visualized at the interface of the desired gradient was centrifuged at 1000 rpm and cells re-suspended in 10 ml of culture medium containing Iscove Modified Dulbecco’s Medium (IMDM, Sigma) supplemented with 1% Penicillin-Streptomycin (P/S, Sigma), 1% Insulin-Transferrin-Sodium Selenite (I/T/S, Sigma), 10% Fetal Bovine Serum (FBS, Sigma) and 10 ng/ml Basic-Fibroblast Growth Factor (b-FGF, Sigma) and seeded in Petri dishes (Corning, USA) placed at 37°C–5% CO_2_ for their amplification. Daily, microscopic observation of cultures allowed the recognition of two different adherent cell populations, one with mesenchymal-like and one with monomorphic blast-like characteristics. This latter population constitutes the so-called Cardiac Progenitor Cells (CSPCs), which exhibit clonogenic growth, multipotency and self-renewing ability, as extensively shown in rodent and human hearts [10], [25]. We used the term Cardiac Stem/Progenitor Cells (CSPCs) because in our typical preparations a consistent fraction of c-kit or Sca-1 positive cells in the heart expressed nuclear transcription factors (GATA-4/MEF2C/GATA-6/ETS-1) indicating cardiovascular lineage commitment [25]. These cells were amplified for several passages and cryo-preserved in aliquots in a medium composed by FBS supplemented with 1% Dimethylsulphoxide (DMSO, Sigma).

#### Co-cultures: CSPCs-cardiomyocytes

In order to evaluate whether and to which extent the functional properties of CSPCs were affected by parenchymal cells of RSV-treated and untreated hearts, control CSPCs (previously expanded from passage 1 to 4) were seeded at a concentration of 35,000/cm^2^ together with ventricular cardiomyocytes (at a concentration of 18,000/cm^2^) isolated from untreated and RSV-treated D1, D3 and D8 hearts. Growth (phospho-histone H3-positive CSPCs; polyclonal rabbit anti-ph-H3, Upstate, Charlottesville, VA, USA) and survival characteristics (TUNEL assay) of CSPCs were analyzed after 72 hours of co-culture.

#### Analysis of conditioned media

The conditioned medium from each co-culture was harvested after 72 hours and evaluated using RayBio Rat Cytokine Antibody Array II purchased from RayBiotech (Norgross, GA, USA). This assay can simultaneously detect the expression level of different cytokines with high specificity (Table 1).

**Table 1:** Cytokine Antibody Arrays.

Activin A	IL-4
Agrin	IL-6
B7-2/CD86	IL-10
beta-NGF	IL-13
CINC-1	Leptin
CINC-2alpha	LIX
CINC-3	L-Selectin
CNTF	MCP-1
Fas Ligand	MIP-3alpha
Fractalkine	MMP-8
GM-CSF	PDGF-AA
ICAM-1	Prolactin R
IFN-gamma	RAGE
IL-1alpha	Thymus Chemokine-1
IL-1beta	TIMP-1
IL-1 R6	TNF-alpha
IL-2	VEGF

Briefly, after treating the membranes with a blocking buffer, 1 ml of conditioned medium was added and incubated at room temperature for 2 hours. The membranes were washed, and 1 ml of primary biotin-conjugated antibody was added and incubated at room temperature for 2 hours. The membranes were then incubated with 2 ml of horseradish peroxidase-conjugated streptavidin at room temperature for 1 hour and subsequently developed by using enhanced chemiluminescence-type solution (Immobilon Western-Millipore) and exposed to Kodak X-Omat AR film. The intensities of signals were quantified by densitometry (software for image capturing and analysis: ImageQuant-Molecular Dynamics). For each spot the net density was determined by subtracting the background gray level. The density of the positive control spots were used to normalize the results from the different membranes.

### Cardiac Anatomy and Morphometry

In 9 C, 9 D1, 14 D1R, 7 D3, 10 D3R, 5 D8 and 8 D8R rats, the abdominal aorta was cannulated, the heart was arrested in diastole with injection of CdCl_2_ solution (100 mM), and the myocardium was retrogradely perfused with 10% buffered formalin solution. The left ventricular chamber was filled with fixative at a pressure equal to the *in vivo* measured systolic pressure. The heart was then excised and placed in formalin solution (10%) for 24 hours.

#### Cardiac anatomy

The right ventricle (RV) and the left ventricle (LV) inclusive of the septum were separately weighed and the volume of the left ventricular myocardium was computed by dividing LV myocardial weight by the specific weight of the tissue (1.06 g/ml). LV chamber length was measured from the apex to the aortic valve. A 1-mm-thick transverse slice was cut from the mid-region of the LV and used to compute LV wall thickness and chamber equatorial diameter (Image Pro-plus, Media Cybernetics, Bethesda, MD, USA, version 7.0). The LV chamber volume was calculated according to the Dodge equation which equalizes the ventricular cavity to an ellipsoid [26]. The slice was then embedded in paraffin and five-micrometer-thick sections were cut and used for morphometric and immunohistochemical analyses.

#### Morphometric analysis

Sections were stained with Masson’s trichrome and analyzed by optical microscopy (magnification 250X) in order to evaluate in the ventricular myocardium: (i) the volume fraction of myocytes, (ii) the volume fraction of interstitial and perivascular fibrosis, and (iii) the numerical density and average cross-sectional area of fibrotic foci. According to a procedure previously described [27], for each section, these analyses were performed in 60 adjacent fields from sub-endocardium, mid-myocardium and sub-epicardium. The measurements were obtained with the aid of a grid defining a tissue area of 0.160 mm^2^ and containing 42 sampling points each of them covering an area of 0.0038 mm^2^.

### Immunohistochemistry

Atrial and LV sections were analyzed to estimate (i) the percentage of apoptotic cells (TUNEL assay), (ii) the numerical density of cells expressing c-kit, the receptor for Stem Cell Factor (rabbit polyclonal anti-c-kit antibody, Santacruz Biotechnology, Santa Cruz, CA, USA), (iii) the relative expression of α-skeletal actin (α-SKA) by using a specific monoclonal antibody against α-SKA (clone 10D2, IgG2a). This antibody has been developed and characterized in the Dept. of Pathology and Immunology, University of Geneva, Switzerland [28]. In five animals of each group, sections were used for immunohistochemistry with anti-α-SKA antibody. Total heart sections were scanned using a fully automated system (Mirax Scan, Zeiss, Oberkochen, Germany). Images were subsequently analyzed and α-SKA positive cardiomyocytes were quantified using the software Metamorph (Molecular Devices, Sunnyvale, CA). Myocardial areas were considered positive to the immunostaining when their pixel intensity values overcame background values. Positive areas were then expressed as percentage of the total heart section.

### Electrophoresis and Immunoblot Assay

In 3 rats for each group, the hearts were excised, and the left and right ventricles were weighed and immediately frozen at −80°C. Western blot assay was used to assess the expression levels of high-mobility group box-1 protein (HMGB-1) as an index of activation of pro-inflammatory signal cascades in ventricular myocardial tissue.

The left ventricular tissue was mechanically fragmented in liquid nitrogen, homogenized in Sample Buffer (62.5 mM Tris-HCl, pH 6.8, 2% sodium dodecyl sulfate (SDS), 10% glycerol, 50 mM DTT, 0.01% bromophenol blue) and boiled for 5 min. For each animal, 50 µg of proteins were separated on 10% polyacrylamide gels and electroblotted on nitrocellulose membranes (Protran, Schleicher & Schuell, Dassel, Germany). Membranes were blocked with 5% milk in TBS-T (Tris-Buffered Saline Tween-20) and were incubated overnight at 4°C with the primary antibody (anti-HMGB1 rabbit polyclonal antibody, Abcam, Cambridge, UK). After washing the membranes, a second incubation was performed for one hour at room temperature with peroxidase conjugated affinity purified goat anti-rabbit secondary antibody (Jackson Immunoresearch Laboratories, West Grove, PA). Peroxidase activity was developed using the ECL Western blotting system (Amersham, Rahn AG, Zürich, Switzerland), according to the instructions of the manufacturer. To determine the expression levels of HMGB-1, blots were scanned and the intensity of the band was quantified by means of the ImageJ Program (NIH, Bethesda, MD, USA). Actin (anti-actin rabbit polyclonal antibody, Sigma) was used as the loading control.

### Statistical Analysis

Power analysis has been performed to evaluate the research design and minimize Type II errors and sample size. Preliminary hemodynamic data obtained in a pilot study have been used. The ANOVA statistical test was used for computing the F value, the significance level, the effect size and the non-centrality parameter (PASW Statistics 18). Then, a-priori power analysis was used (G*Power Version 3.1.2; Franz Faul, Kiel University, Germany) by setting α = 0.01 and β = 0.05 (power 1-β = 0.95) in accordance with the suggestion of Cohen [29], to estimate total sample size and the number of measurements required for each group. Statistics of variables included mean±standard error (SE), Multifactorial analysis of variance (1 way ANOVA followed by Bonferroni’s test or Dunnett’s test when appropriate). Statistical significance was set at p<0.05.

## Results

### Glucose Blood Levels and Body Weight

At the beginning of the experimental protocol, glucose blood levels and body weight ranged respectively between 102–109 mg/dl and 301–445 g, in the entire rat population. After STZ injection, glucose blood levels were higher than those measured in C group, at every time points of observation (Table 2). In diabetic animals, body weight decreased by 15% during the first week of hyperglycemia to remain essentially constant until 8 weeks (Table 3). RSV treatment did not significantly affect both glucose blood levels and body weight (Tables 2, 3).

**Table 2:** Glucose blood levels (mg/dl).

	*D1*	*D1R*	*D3*	*D3R*	*D8*	*D8R*
Day 0	110±10	108±7	111±6	115±11	101±9	105±8
Day 2	371±12*	404±21*	399±18*	380±7*	370±11*	433±13*
Day 7	454±16*	443±13*	527±30*	492±31*	498±43*	480±22+
Day 14			554±21*	527±17*	510±22*	505±21*
Day 21			442±36*	515±18*	520±25*	525±23*
Day 28					528±25*	568±11*
Day 35					527±21*	555±18*
Day 42					526±20*	546±27*
**Day 56**					529±18*	554±20*

Values are mean ± SE of glucose blood levels weekly measured in diabetic (D) and RSV-treated diabetic rats (DR)

* p<0.01 significant differences from control animals.

**Table 3:** Body weight (g).

	*D1*	*D1R*	*D3*	*D3R*	*D8*	*D8R*
Day 0	382±7	371±5	363±10	374±8	353±3	348±4
Day 7	340±2	334±11	341±13	345±10	335±4	331±6
Day 14			334±15	319±10	342±6	333±6
Day 21			335±12	327±8	340±7	336±7
Day 28					332±10	310±5
Day 35					332±9	312±6
Day 42					331±8	314±7
**Day 56**					346±7	311±4

Values are mean ± SE of body weight weekly measured in diabetic (D) and RSV-treated diabetic rats (DR).

### Hemodynamics

**Figure 1:**
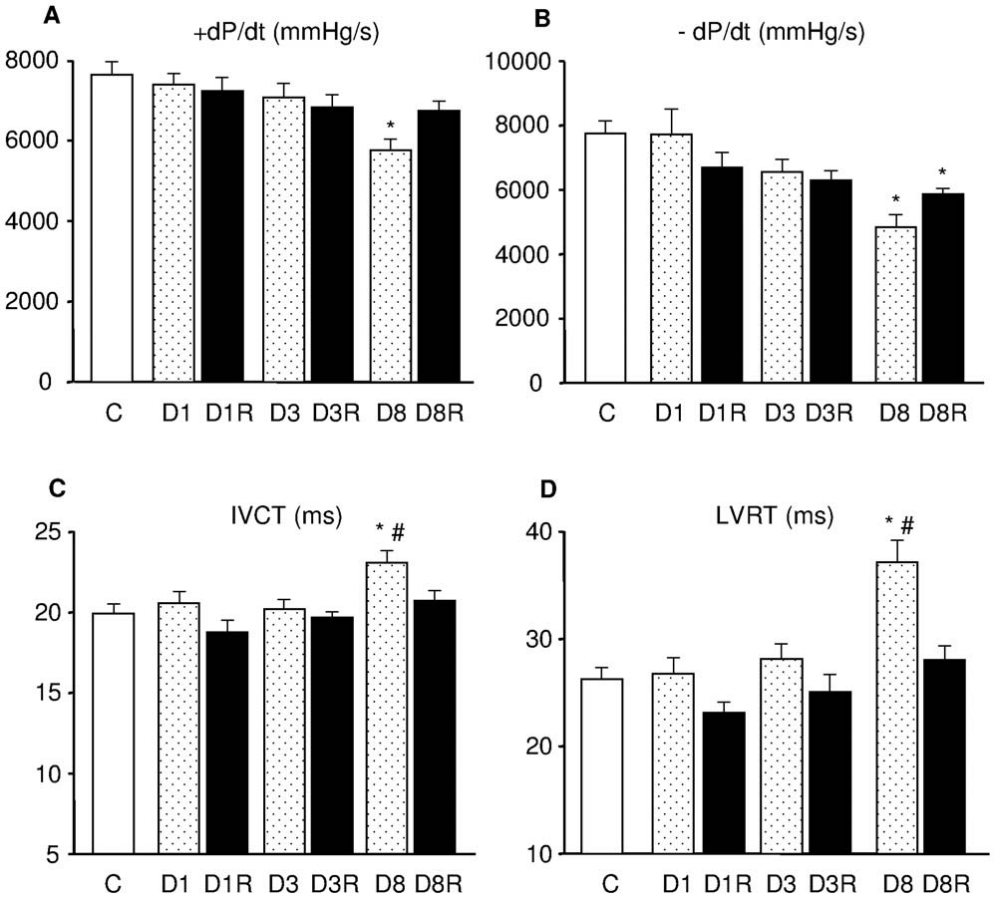
Hemodynamic measurements. Mean values ± SE of: A) maximum rate of ventricular pressure rise (+dP/dt), B) maximum rate of ventricular pressure reduction (-dP/dt), C) isovolumic contraction time (IVCT), and D) LV relaxation time (LVRT), measured in control rats (C) and untreated or RSV-treated diabetic rats after 1 (D1, D1R), 3 (D3, D3R) and 8 (D8, D8R) weeks of hyperglycemia. *p<0.01: significant differences vs. C; # p<0.05: significant differences between D8 and D8R.

Heart rate measured under anesthesia was similar in all experimental groups (heart rate ranging from 195 and 220 bpm, in the different groups). In comparison with C rats, all diabetic groups exhibited significantly lower values of systolic LV pressure (range: 103–112 mmHg vs. 100–150 mmHg measured in C) while end-diastolic pressure was unchanged (range: 5–9 mmHg). Marked signs of systolic and diastolic ventricular dysfunction developed after 8 weeks of diabetes, including a significant reduction in ±dP/dt (Figure 1 A–B), associated with a prolongation of both isovolumic contraction time (IVCT) and relaxation time (LVRT) (Figure 1 C–D). In D8R group, RSV treatment abolished the negative impact of diabetes on LV hemodynamics and all parameters reached control values with the exception of the rate of pressure decline during relaxation (-dP/dt) (Figure 1 B).

### Cell Mechanics and Calcium Transients

Globally, 638 isolated ventricular myocytes were used for cell mechanics (125 from C hearts, 85 from D1, 80 from D3, 83 from D8, 111 from D1R, 95 from D3R and 59 from D8R). In 30 cells of each experimental group, calcium transients were simultaneously recorded. The average diastolic sarcomere length was comparable in all groups (average sarcomere length equal to 1.71±0.002 µm).

**Figure 2:**
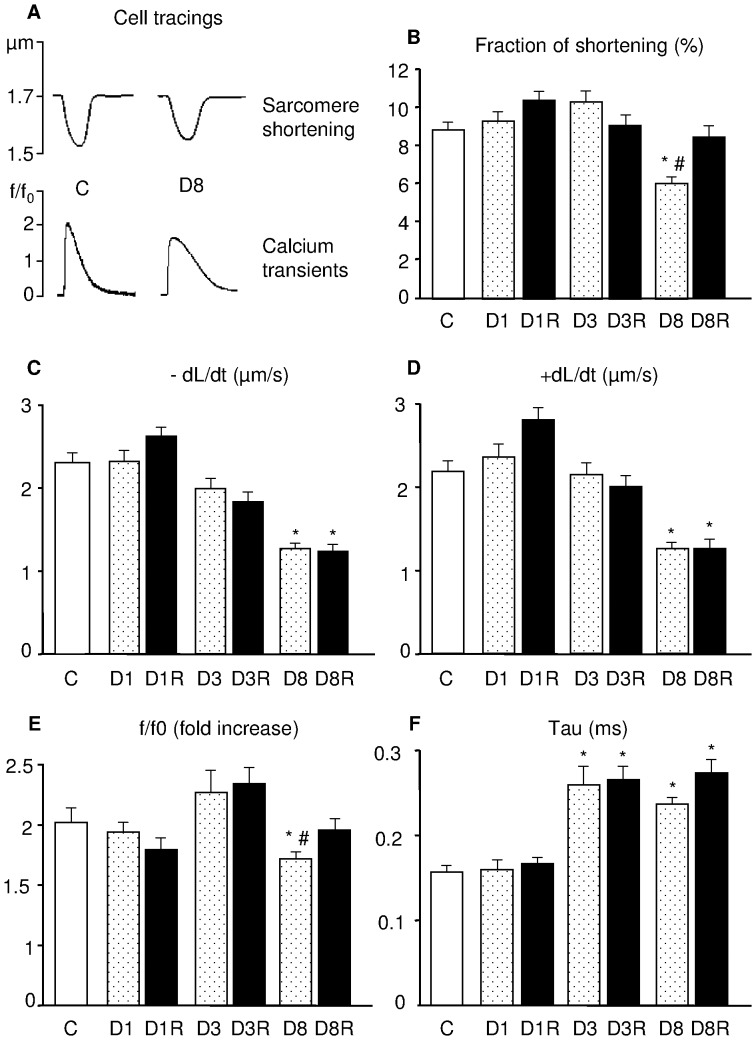
Cell mechanics and intracellular calcium transients. A) Representative examples of sarcomere shortening and corresponding calcium transients (normalized tracings: fold increase) recorded from C and D8 ventricular myocytes. In bar graphs, the mean values ± SE of: B) sarcomere fraction of shortening, C) maximal rate of shortening (-dL/dt), D) maximal rate of relengthening (+dL/dt), E) calcium transient amplitude expressed as peak fluorescence normalized to baseline fluorescence (f/f0), and F) time constant of the intracellular calcium decay (Tau), measured in control myocytes (C) and untreated or RSV-treated diabetic cells (D and DR, respectively), after 1, 3 and 8 weeks of hyperglycemia. * p<0.01: significant differences vs. C; # p<0.05: significant differences between D8 and D8R.

In accordance with the *in vivo* recorded hemodynamic data, the mechanical properties of ventricular myocytes from D1 and D3 diabetic hearts were globally preserved (Figure 2 B–D), except for the lower rate of intracellular calcium clearing (TAU) in D3 (Figure 2 F). Conversely, a definite worsening of cell mechanics occurred in D8 (Figure 2 A–D). In comparison with C cells, D8 cardiomyocytes exhibited a reduced fraction of shortening associated with a significant decrease in the maximal rate of shortening (-dL/dt) and relengthening (+dL/dt) (p<0.01; Figure 2 B–D). The impaired contractility in D8 cells was accompanied by a significant decrease in calcium transient amplitude (f/f0) (−15%, p<0.01; Figure 2 E) and a prolonged TAU (+50%, p<0.01; Figure 2 F). The progressive impairment of cardiomyocyte contractile efficiency produced by diabetes was partially improved by RSV treatment as shown by the recovery of fraction of shortening and f/f0 (Figure 2 B, E).

### Effects of Diabetes and RSV on Cardiac Anatomy and Myocardial Tissue

**Figure 3:**
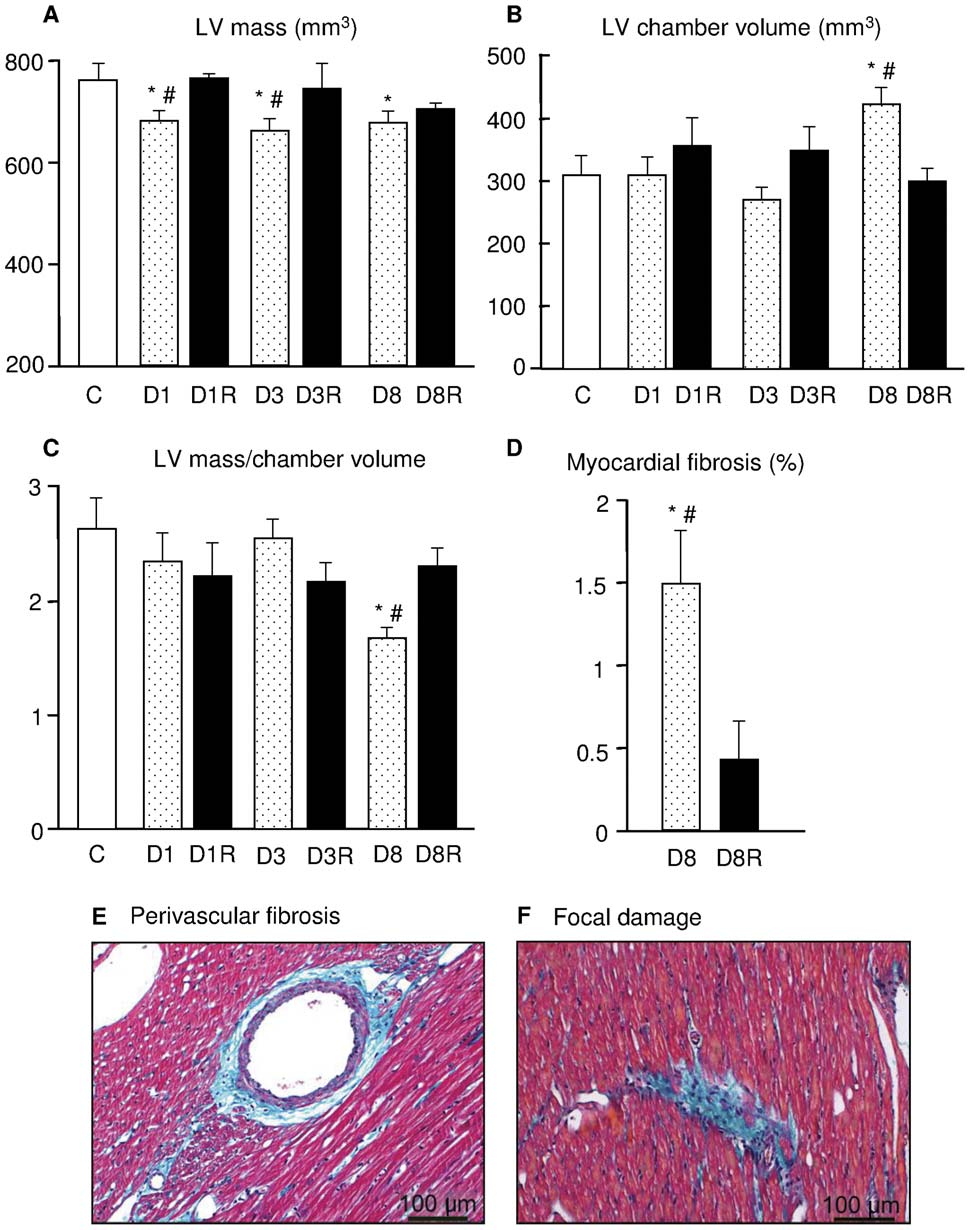
Cardiac anatomy and tissue morphometry. In A–C graphs the mean values ± SE of the left ventricular (LV) geometrical properties measured in the different experimental groups: A) LV mass, B) LV chamber volume, and C) LV mass to chamber volume ratio. In D) mean values ± SE of the volume fraction of fibrosis morphometrically determined in D8 and D8R groups. E–F: sections of LV mid-myocardium stained with Masson’s trichrome showing in greenish blue the fibrotic tissue in a D8 rat heart. E: accumulation of collagen in perivascular space. F: focal damage characterized by the presence of collagen and inflammatory cell infiltrate. Scale bars = 100 µm. * p<0.05: significant differences vs. C; # p<0.05: significant differences vs. the corresponding RSV-treated group.

Diabetes induced a marked loss of left ventricular mass in D1, D3 and D8 untreated hearts (p<0.05; Figure 3 A), in the absence of significant changes in individual cardiomyocyte size (average cardiomyocyte surface area: approximately 1,400**µm^2^, data not shown). A significant dilation of LV chamber was observed only after 8 weeks of diabetes (Figure 3 B) leading to a marked reduction in mass-to-chamber volume ratio in D8 group (Figure 3 C). These unfavorable anatomical changes produced by diabetes were reversed by RSV administration (Figure 3 A–C).

**Figure 4:**
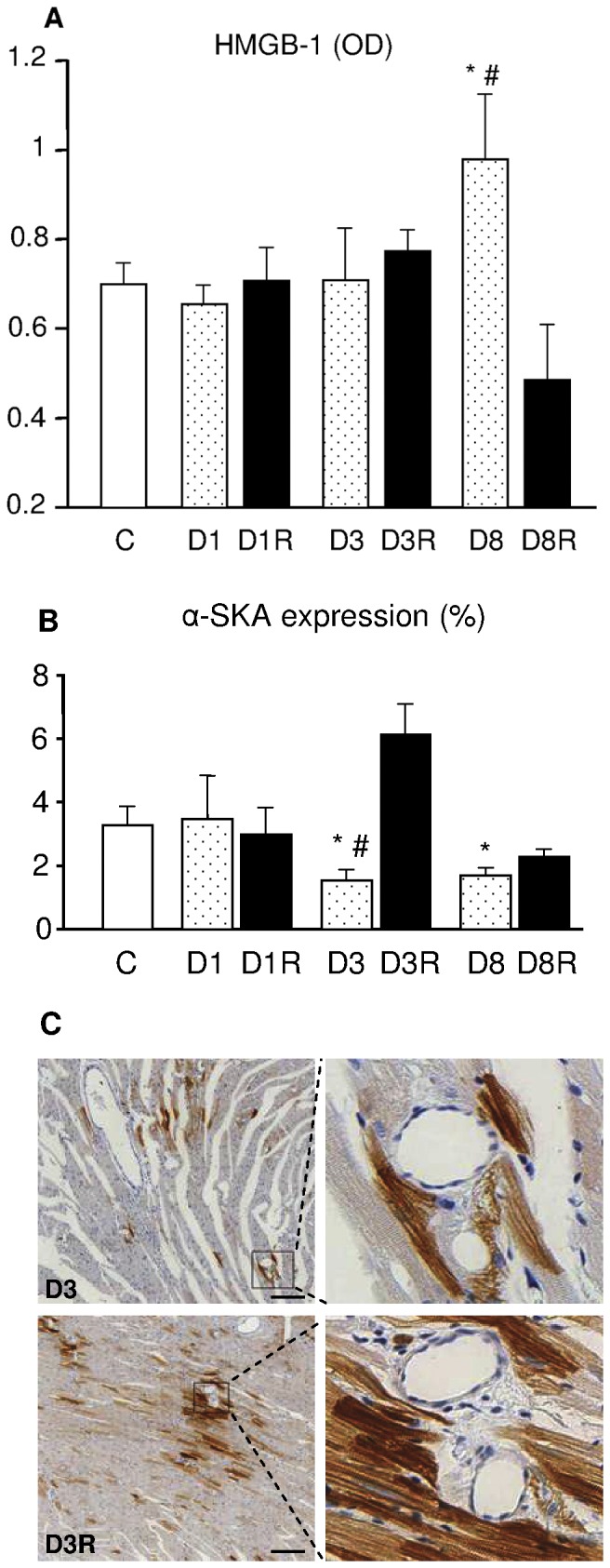
HMGB-1 and alpha-SKA expression in left ventricular myocardium. A–B: Expression levels of A) high mobility group box-1 protein (HMGB-1) and B) α-SKA, in left ventricular myocardial tissue. Data are reported as mean ± SE. * p<0.01: significant differences vs. C. # p<0.05: significant differences vs. the corresponding RSV-treated group. C: sections of D3 (upper panels) and D3R (lower panels) LV myocardium stained with anti α-SKA. Black squares inscribe an area shown at higher magnification in corresponding adjacent panels. Scale bar = 100 µm.

The total amount of collagen accumulation was negligible and similar to control ventricles in RSV-treated and untreated diabetic hearts during the first 3 weeks of hyperglycemia. In D8 group, although myocardial damage was still limited, the total amount of fibrosis in the myocardium was significantly increased in comparison with C (Figure 3 D). Perivascular fibrosis was observed in association with small foci of reparative fibrosis characterized by inflammatory cellular infiltrates (Figure 3 E–F), more evident in the mid-myocardium. The activation of pro-inflammatory signal cascades was confirmed by the significant increase in HMGB-1 expression in D8 group, as revealed by Western blotting assay (Figure 4 A). Cardiac damage and inflammation signals were completely prevented by RSV administration (Figure 3 D, 4 A).

Significantly lower values of α-SKA expression were also detected in diabetic cardiomyocytes, after 3 and 8 weeks of hyperglycemia (−50% on average in both groups vs C; Figure 4 B–C). This reduction was counteracted by RSV-treatment in D3R group (Figure 4 B–C) while the effect of RSV was less evident at the later time point (D8R group).

### Effects of RSV on Diabetes-induced Cardiomyocyte and Endothelial Cell Apoptosis

**Figure 5:**
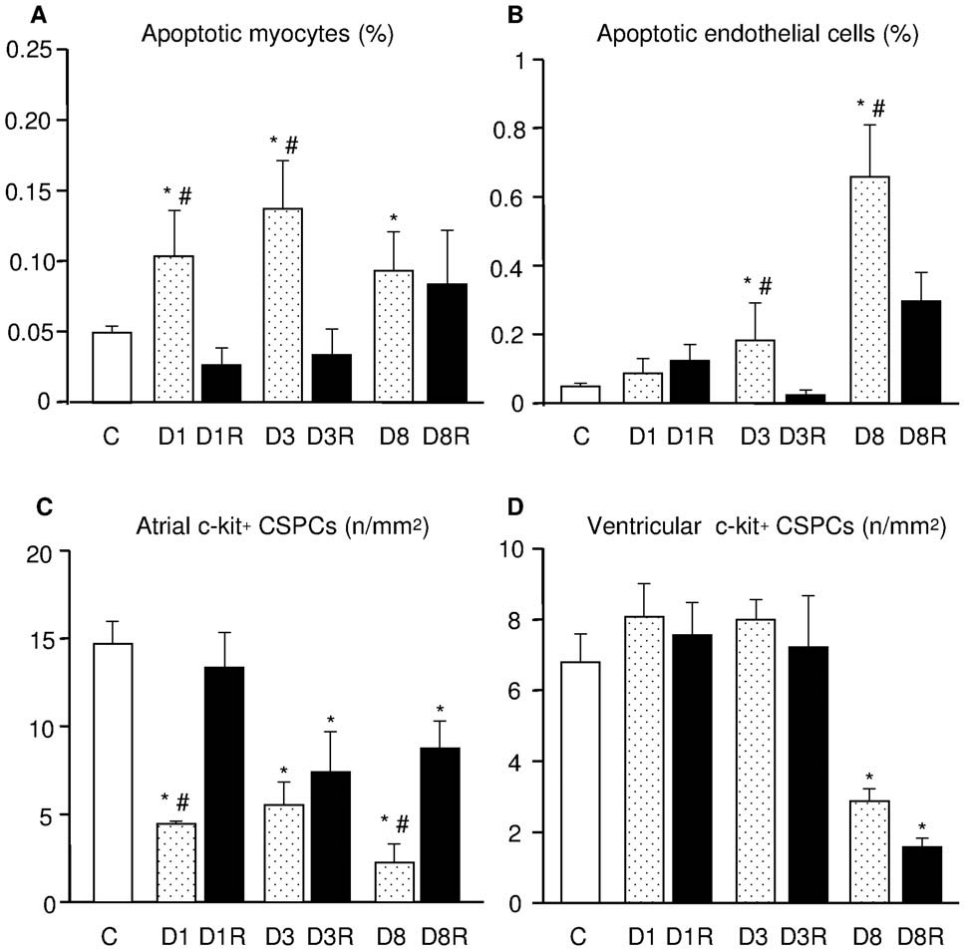
Effects of diabetes and RSV on myocardial cell apoptosis and CSPC number. Bar graphs illustrate the percentage of apoptotic cardiomyocytes (A) and endothelial cells (B), and the numerical density of atrial (C) and ventricular (D) c-kit+ CSPCs. Data are expressed as mean values ± SE. * p<0.01: significant differences vs. C. # p<0.05: significant differences vs. the corresponding RSV-treated group.

To determine ongoing apoptotic cell death, tissue sections of the LV myocardium were subjected to the TUNEL assay. In comparison with control hearts, the percentage of apoptotic cardiomyocytes increased with diabetes at all time points of observation (Figure 5 A) while apoptosis in endothelial cells increased from 3 to 8 weeks (Figure 5 B). Overall, RSV treatment was able to reduce by 65% to 85% the incidence of apoptotic cell death (Figure 5 A–B) although, after 8 weeks of diabetes, a marked protective effect was maintained only on endothelial cells (Figure 5 B).

### Effects of Diabetes and RSV Treatment on CSPCs

#### Tissue analysis

To evaluate the effects of diabetes and RSV treatment on the numerical density and tissue distribution of CSPCs, sections of the atria and LV from all experimental groups were immunostained for the detection of c-kit, the receptor of Stem Cell Factor, representing the most preserved stem cell antigen among different tissues of different species [30].

As expected, the quantitative analysis documented that in control hearts the number of c-kit-positive CSPCs was 2.5 fold higher in the atria with respect to the LV supporting the notion of preferential sites of accumulation of cardiac primitive cells [31]. Diabetes significantly reduced the density of CSPCs in the atrial appendages at all time points (Figure 5 C). Conversely, the deleterious effect of diabetes on left ventricular CSPCs was detected only after 8 weeks of hyperglycemia (Figure 5 D). RSV administration significantly attenuated the early and late dramatic depletion of CSPCs from the atrial sites of storage (Figure 5 C), although was not able to counteract the late diabetes-induced loss of progenitor cell compartments in the working left ventricle (Figure 5 D).

#### *In vitro* analysis

It is well established that, especially at the level of niches, stem cell function is tightly regulated by environmental factors [32]. Thus, we determined *in vitro* whether the negative impact of diabetes and the protective action of RSV on CSPCs could be attributed to changes in myocardial environment. Specifically, the effect of diabetic cardiomyocytes, isolated from RSV-treated and untreated diabetic hearts, on the magnitude of CSPCs apoptotic death (TUNEL) and proliferation (Ph-H3) was analyzed.

**Figure 6:**
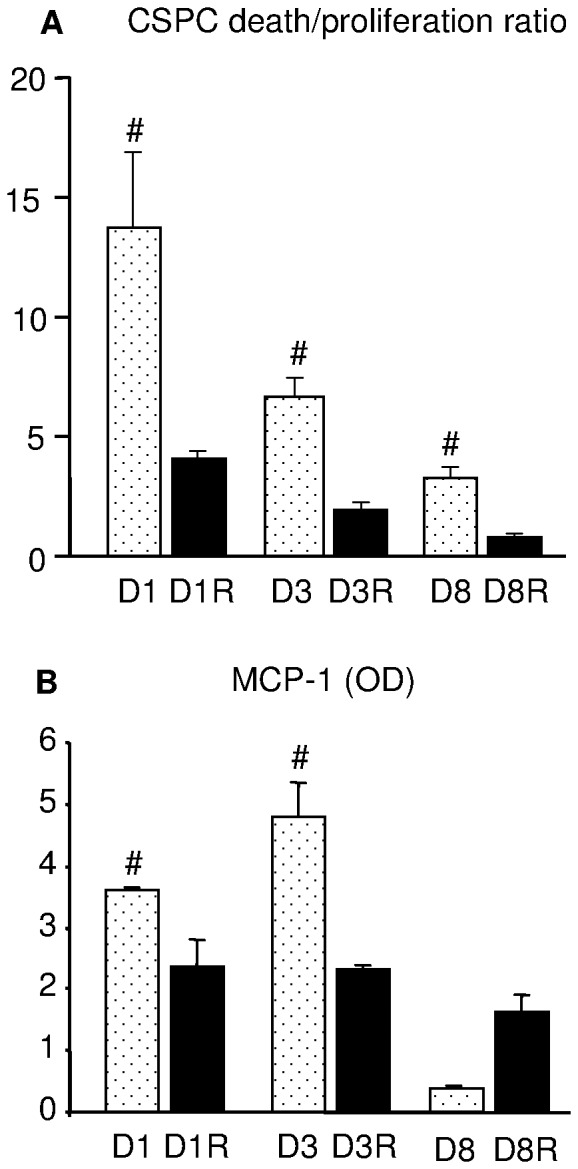
Co-cultures of CSPCs and cardiomyocytes. A: death to proliferation ratio of CSPCs co-cultured with untreated or RSV-treated diabetic cardiomyocytes; B: concentration of the pro-inflammatory cytokine monocyte chemotactic protein-1 (MCP-1) in media harvested from co-cultures at different experimental time points. OD: optical density. Data are expressed as mean values ± SE. # p<0.05: significant differences vs. co-cultures containing RSV-treated myocytes.

CSPCs co-cultured with D1, D3 or D8 diabetic cardiomyocytes exhibited a higher rate of death associated with a lower proliferation capacity leading to an increased death-to-proliferation ratio, in comparison with CSPCs co-cultured with RSV-treated cardiomyocytes, at all time points of observation (Figure 6 A). However, no linear relationship with the duration of diabetes was observed on this parameter. These *in vitro* observations suggest that the diabetic “milieu” impairs CSPC turnover which can be reversed by RSV. To investigate this phenomenon, we determined the relative concentrations of several cytokines in the media harvested from each co-culture condition (Table 1).

The presence of cardiomyocytes isolated from D1 and D3 untreated diabetic hearts mainly resulted in a higher concentration of the monocyte chemotactic protein-1 (MCP-1) as compared with the conditioned media collected from co-cultures containing RSV-treated cardiomyocytes (D1R, D3R; Figure 6 B). These data suggest that the inflammatory and pro-apoptotic bulk produced by diabetes can be attenuated by chronic RSV administration.

## Discussion

The most important finding of the present study is the demonstration of positive effects of early RSV-treatment on “myocardial diabetic milieu” leading to a reduced inflammatory state and attenuation of cell loss. The improved tissue environment positively interferes with CSPC survival and functional properties, resulting in a decreased ventricular remodeling produced by diabetes. The functional counterpart of these effects consisted in the recovery of cardiac hemodynamics associated with a partial restoration of cardiomyocyte mechanical properties.

Although it is well accepted clinically that diabetes causes myocardial dysfunction through accelerated atherosclerosis and hypertension, there is evidence that DCM is a specific clinical condition that occurs in diabetic patients in the absence of coronary atherosclerosis and hypertension [2]. This suggests that hyperglycemia and associated metabolic disturbances may “per-se” negatively affect cardiac structure and function triggering alterations in myocardial substrate which develop independently of vascular disease. The STZ-rat model used in this study is particularly relevant in examining the effects of hyperglycemia alone on myocardial morpho-functional properties, in the absence of hyperinsulinemia and atherosclerosis [2].

DCM consists of a series of structural and functional changes, including chronic loss of myocytes and vascular cells leading to a decrease in muscle mass, chamber dilation, altered extracellular matrix and impaired systolic and diastolic ventricular function. Accumulating evidence supports the concept that a “cardiac stem cell compartment disease” plays an important role in the pathophysiology of DCM [9]. Oxidative stress and inflammation are enhanced with diabetes and can induce defects in both growth and survival of CSPCs with a consequent imbalance between cell death and cell replacement, favoring the onset of DCM and its progression towards heart failure [33]. Thus, the preservation of CSPC compartment can contribute to counteract the negative impact of diabetes on the myocardium.

Despite several experimental studies suggesting that antioxidants reduce diabetic cardiovascular complications, results from clinical trials have been disappointing [4], [34] probably because traditional antioxidants have a scavenging action on already formed ROS but limited effects on their intracellular production [34]. Here, we tested the hypothesis that early administration of RSV can be efficient in preventing DCM. In comparison with conventional antioxidant compounds, RSV holds great promise in the treatment of cardiovascular complications of diabetes due to its well known effects on several target molecules resulting in reduced inflammation and intracellular ROS production, decreased rate of apoptotic cell death and increased cellular expression of survival proteins. In addition, RSV was shown to enhance stem cell survival and cardiac regeneration in ischemic heart [13], [20], [35]–[39].

RSV, as other natural phenolic compounds, at low doses promotes antioxidant effects while at high doses can have a dose-dependent pro-oxidant role followed by cell damage, p-Akt down-regulation and apoptosis [40]. In the present study, the RSV dose of 2.5 mg/Kg/day was selected on the basis of (i) experimental and clinical data reported in the literature showing that the most suitable doses of the compound leading to cardio-protection range between 1 mg/Kg/day to 10 mg/Kg/day [17], [41]–[43], (ii) available data on pharmacokinetics and metabolism of RSV [44]–[45], and (iii) the results obtained in the pilot study reported in the Supporting Information (Text S1), showing that 2.5 mg/Kg/day and 5 mg/Kg/day led to comparable positive effects on cardiac function, while the lower dose (1 mg/Kg/day) was less effective (Figure S1).

Increased oxidative stress is the common element to all pathways leading to cell dysfunction and apoptosis in diabetes [2], [33], [46]. Hyperglycemia affects several other mechanisms leading to the generation of advanced glycation end-products (AGEs) and activation of diacylglycerol (DAG)-protein kinase C (PKC), resulting in chronic inflammation [47]–[48]. This unfavorable tissue environment, besides inducing myocyte/endothelial cell damage, can impair mobilization, survival and proliferation of CSPCs. In accordance with previous reports [9], [11], we found a progressive reduction in CSPC density both in atrial storage and ventricular myocardial tissue of diabetic rats, partially reverted by RSV administration. The critical role played by the adverse tissue environment on CSPC survival and proliferation was also supported by data obtained in the co-cultures of healthy CSPCs and cardiomyocytes isolated from diabetic rats.

Co-culture conditioned media showed a high concentration of the pro-inflammatory cytokine monocyte chemotactic protein-1 (MCP-1). It has been recently shown [49] that hyperglycemia-induced expression of MCP-1 and MCP-1-induced protein (MCPIP) leads to ROS production, endoplasmic reticulum stress, autophagy and cardiomyocyte death, thus playing a critical role in the pathophysiological progression of diabetic cardiomyopathy [49]. Systemic RSV treatment resulted in a reduced activation of pro-inflammatory pathways, as indicated by the lower levels of MCP-1 in the co-culture supernatant. A reduced tissue inflammation was also confirmed by the decreased inflammatory cell infiltration and lower expression of HMGB-1 in the ventricular myocardium, after 8 weeks of diabetes. HMGB-1, besides its well known nuclear function in eukaryotic cells, possesses an extracellular role as pro-inflammatory cytokine [50]. The fact that peak values of MCP-1 levels were measured in co-culture media after 3 weeks of diabetes while HMGB-1 peaked at tissue level after 8 weeks may just reflect a different temporal implication of the two cytokines in the inflammatory process. Oxidative stress results in enhanced MCP-1 expression triggering monocytes/neutrophil infiltration (49), thus playing an important role in the early phase of inflammation. The increased HMGB-1, released in a passive way from necrotic cells or in an active way by macrophages [51]–[52], acts as a late mediator of inflammation [53]. Our findings are in accordance with these observations in that: a) the increased oxidative stress, the first negative impact of hyperglycemia, leads to a marked apoptotic cell death [11], in the absence of inflammatory infiltrate, and potentially triggers the increase in MCP-1 expression; 2) infiltrating inflammatory cells and cardiomyocyte necrosis resulting in extracellular release of HMGB-1 occurred in the LV only at the late time point of diabetes (D8). Moreover, the assessment of the expression of MCP1 was carried out in co-cultures of cardiomyocytes with multipotent cells where only apoptotic cell death occurred. The decrease in HMGB-1 expression in treated animals after 8 weeks of diabetes (D8R) is allegedly due to the RSV-induced reduction of both cardiac cell death and recruitment of inflammatory cells.

Oxidative stress and tissue inflammation represent important factors leading to lesions of small coronary arteries and appearance of the perivascular/interstitial fibrosis occurring in diabetic patients and in experimental models of diabetes [33]. The antioxidant effect of RSV associated with the decrease in the pro-inflammatory “milieu” may account for the preservation of ventricular mass and the decreased fibrotic damage observed in treated hearts, resulting in attenuation of cardiac remodeling and improved myocardial function. In addition, it should be outlined that, independently of the amount of loss in LV mass which was comparable in all untreated groups (D1, D3 and D8), a significant decrease in contractile efficiency was observed only in D8 animals, both at the organ and cellular levels. This behavior can be explained by considering that (a) significant alterations in the extracellular matrix (myocardial fibrosis and inflammation), affecting LV mechanical efficiency at the organ level, appeared at the later time point of observation (D8) whereas they were absent in D3 and D1; and 2) intracellular calcium handling was significantly modified only in D8 isolated cardiomyocytes leading to an impaired cellular contractile efficiency, while no significant changes were observed at earlier time points (D1 and D3). Taken together these findings suggest that, at these stages of diabetes, the functional efficiency of individual cells and myocardial restructuring play a more relevant role than LV mass itself in determining cardiac performance.

Our data also indicate that diabetes affects the expression of α-SKA in ventricular cardiomyocytes. During adult life, α-CA is the main isoform present in myofibrils whereas α-SKA distribution appears focal and represents about 5% of the total actin [22]. Previous studies showed that a higher α-SKA content is associated with faster heart dynamics [54] and may constitute a compensation mechanism to maintain crossbridge turnover rate and achieve a high degree of myocardial contractility in pressure-overload cardiac hypertrophy [27]. Here, we found a significant reduction in α-SKA levels after 3 and 8 weeks of hyperglycemia while RSV treatment produced a recovery of α-SKA expression. Although this effect was much more evident after 3 weeks of diabetes and decreased with time, the early increment in α-SKA levels might contribute to maintain a proper answer to increased workload with a concurrent recovery of hemodynamic performance and cardiomyocyte mechanical properties during contraction, as observed at 8 weeks of diabetes.

Our results support the assumption that early RSV treatment preserves the numerical density and functional abilities of both mature cardiac cells and atrial CSPC storage, improves cardiac microenvironment and reduces unfavorable ventricular remodeling. These positive effects of RSV led to an almost complete recovery of global ventricular hemodynamics and to a partial restoration of cardiomyocyte function at cellular level. Two mechanisms underlying the cardioprotective effects of early RSV treatment have been identified: (i) the reduced levels of pro-inflammatory cytokines in the myocardial environment, as demonstrated in vitro and at the tissue level, and (ii) the increase in sarcomeric alpha-SKA expression which can contribute to maintain contractile properties of ventricular myocytes. However, further studies are required to achieve more mechanistic insights on the molecular pathways involved in RSV-mediated prevention of precocious CSPC aging, CSPC niche depletion, and cardiomyocyte dysfunction, in the diabetic heart. It is also conceivable that combining RSV administration with traditional drugs that reduce metabolic derangements and glucose blood levels, and strategies aimed at enhancing RSV bioavailability [44], [55] can increase and prolong the effects of the compound.

In conclusion, we demonstrated that early administration of RSV can efficiently prevent the occurrence of DCM phenotype in STZ-induced diabetes. This important finding deserves careful consideration in future studies aimed at evaluating RSV as an adjuvant therapeutic option in the treatment of DCM at later stages of the disease.

## Supporting Information

Figure S1 **Hemodynamic measurements.** Mean values ± SE of: A) maximum rate of ventricular pressure rise (+dP/dt), B) maximum rate of ventricular pressure reduction (-dP/dt), C) isovolumic contraction time (IVCT), D) LV relaxation time (LVRT), and E) total contraction-relaxation time, measured in control rats (C) and untreated (D8) or treated diabetic rats with different low doses of RSV: 1 mg/Kg/day (D8 R_1 mg), 2.5 mg/Kg/day (D8 R_2.5 mg), and 5 mg/Kg/day (D8 R_5 mg). *p<0.05: significant differences vs. C; 1-way ANOVA (post-hoc analysis by Bonferroni test).
(TIF)

Table S1 **Urine and plasma concentration of RSV metabolites.** Range and median values of RSV-metabolite concentrations in urine and plasma samples of treated diabetic rats. For each group, the number of animals used for the analysis is indicated between brackets, in the first column. In each animal, concentrations of RSV metabolites were assayed in duplicate (urine) or quadruplicate (plasma). NQ  =  below the limit of quantification.
(DOC)

Text S1 **Detailed description of the preliminary experiments performed to (i) verify dose-dependent effects of long-term administration of RSV on functional parameters, and (ii) select the most suitable dose of the compound.**
(DOC)
