# Supplementary figures and images for: Resveratrol Treatment Reduces Cardiac Progenitor Cell Dysfunction and Prevents Morpho-Functional Ventricular Remodeling in Type-1 Diabetic Rats
Source: PLoS One. 2012 Jun 29;7(6):e39836. doi: 10.1371/journal.pone.0039836 (PMC3387239; doi:10.1371/journal.pone.0039836)

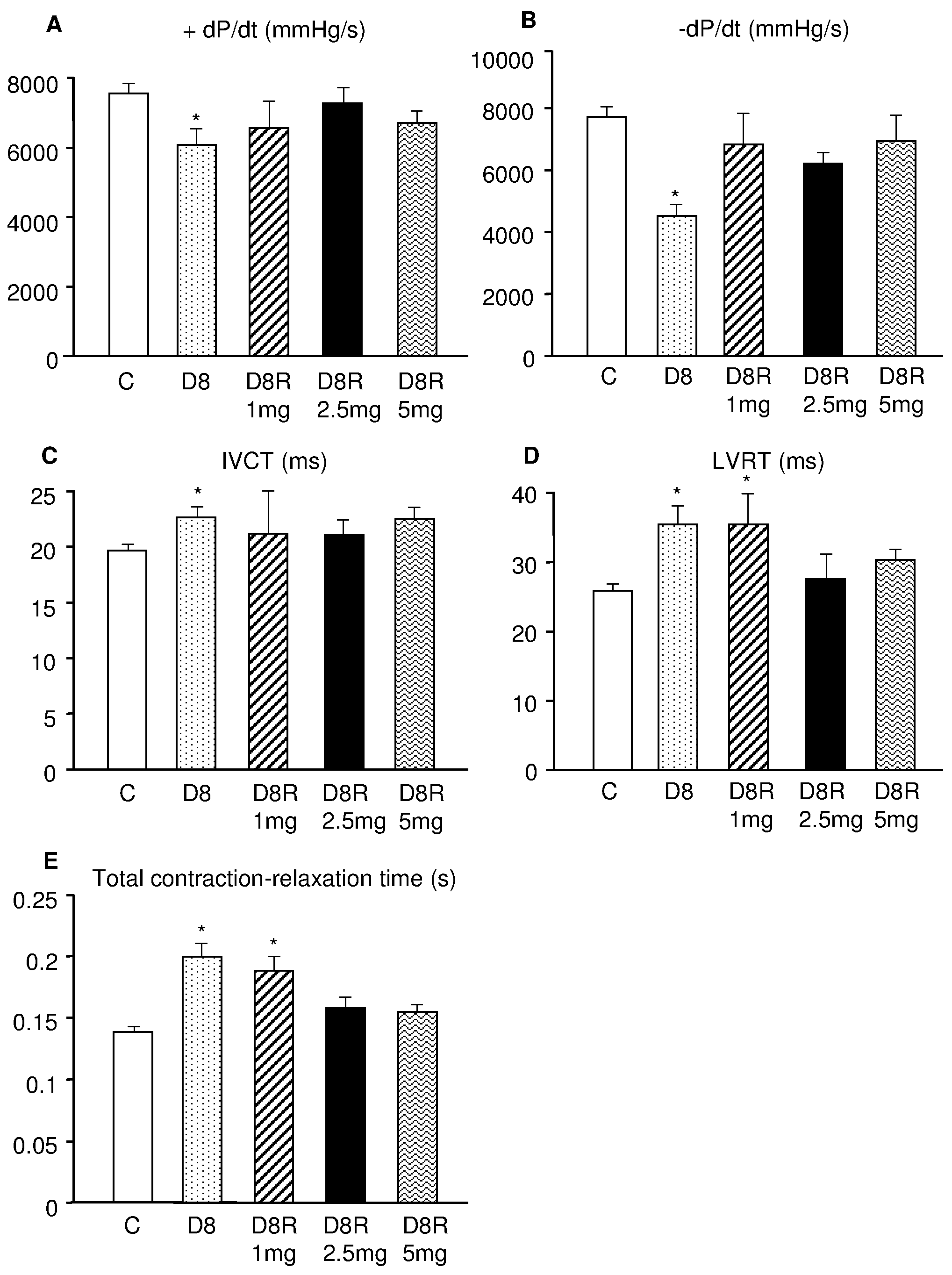

Supplement: Figure S1 — Hemodynamic measurements. Mean values ± SE of: A) maximum rate of ventricular pressure rise (+dP/dt), B) maximum rate of ventricular pressure reduction (-dP/dt), C) isovolumic contraction time (IVCT), D) LV relaxation time (LVRT), and E) total contraction-relaxation time, measured in control rats (C) and untreated (D8) or treated diabetic rats with different low doses of RSV: 1 mg/Kg/day (D8 R_1 mg), 2.5 mg/Kg/day (D8 R_2.5 mg), and 5 mg/Kg/day (D8 R_5 mg). *p<0.05: significant differences vs. C; 1-way ANOVA (post-hoc analysis by Bonferroni test). (TIF) [file pone.0039836.s001.tif]
