# Supplementary material for: Resveratrol Treatment Reduces Cardiac Progenitor Cell Dysfunction and Prevents Morpho-Functional Ventricular Remodeling in Type-1 Diabetic Rats
Source: PLoS One. 2012 Jun 29;7(6):e39836. doi: 10.1371/journal.pone.0039836 (PMC3387239; doi:10.1371/journal.pone.0039836)
Supplement: Table S1 — Urine and plasma concentration of RSV metabolites. Range and median values of RSV-metabolite concentrations in urine and plasma samples of treated diabetic rats. For each group, the number of animals used for the analysis is indicated between brackets, in the first column. In each animal, concentrations of RSV metabolites were assayed in duplicate (urine) or quadruplicate (plasma). NQ = below the limit of quantification. (DOC) [file pone.0039836.s002.doc]

**Table S1. Urine and plasma concentration of RSV metabolites**

| **Groups** | **Urine (nmol/l)** | | **Plasma (nmol/l)** | |  |
| --- | --- | --- | --- | --- | --- |
|  | **RSV-sulfate** | **RSV-glucuronide** | **RSV-sulfate** | **RSV-glucuronide** |  |
| D8R_1mg  (n=2) | 7-78 (n=4)  Median: 41 | 351-4663 (n=4)  Median: 1985 | 16-181 (n=8)  Median: 60 | 14-24 (n=8)  Median: 7 | |
| D8R_2.5mg (n=2) | 104-119 (n=4)  Median: 117 | 3815-4487 (n=4)  Median: 4402 | 384-449 (n=8)  Median: 417 | NQ |  |
| D8R_5mg  (n=4) | 19-932 (n=8)  Median: 34 | 6593-18383 (n=8)  Median:8888 | 126-1078 (n=16)  Median: 495 | 9-45 (n=16)  Median:14 |  |

Range and median values of RSV-metabolite concentrations in urine and plasma samples of treated diabetic rats. For each group, the number of animals used for the analysis is indicated between brackets, in the first column. In each animal, concentrations of RSV metabolites were assayed in duplicate (urine) or quadruplicate (plasma). NQ = below the limit of quantification.
