# Supplementary material for: Resveratrol Treatment Reduces Cardiac Progenitor Cell Dysfunction and Prevents Morpho-Functional Ventricular Remodeling in Type-1 Diabetic Rats
Source: PLoS One. 2012 Jun 29;7(6):e39836. doi: 10.1371/journal.pone.0039836 (PMC3387239; doi:10.1371/journal.pone.0039836)
Supplement: Text S1 — Detailed description of the preliminary experiments performed to (i) verify dose-dependent effects of long-term administration of RSV on functional parameters, and (ii) select the most suitable dose of the compound. (DOC) [file pone.0039836.s003.doc]

**SUPPORTING INFORMATION**

**Resveratrol Treatment Reduces Cardiac Progenitor Cell Dysfunction and Prevents Morpho-Functional Ventricular Remodeling in Type-1 Diabetic Rats.**

Francesca Delucchi^1^, Roberta Berni^1^, Caterina Frati^2^, Stefano Cavalli^2^, Gallia Graiani^2^, Roberto Sala^3^, Christine Chaponnier^4^, Giulio Gabbiani^4^, Luca Calani^5^, Daniele Del Rio^5^, Leonardo Bocchi^1^, Costanza Lagrasta^2†^, Federico Quaini^6†^, Donatella Stilli^1†^.

**Corresponding author**:

Donatella Stilli

Dipartimento Biologia Evolutiva e Funzionale, Università di Parma, Parma, Italy

e-mail: donatella.stilli@unipr.it

**SELECTION OF RESVERATROL (RSV) DOSE**

A cardio-protective role of RSV, without harmful effects, was shown in many experimental studies in which different low doses of the compound have been used, ranging from 1 mg/Kg to 10 mg/Kg per day [1, 2-4]. In comparison with most previous studies, here we planned a chronic RSV treatment of diabetic animals, lasting until 8 weeks. Thus, as described below, we performed preliminary experiments to (i) verify dose-dependent effects of long-term administration of the compound on functional parameters, and (ii) select the most suitable dose.

Animals and experimental protocol. In the pilot study, 21 male STZ diabetic rats, aged 12-14 wk, were used. Animals were studied after 8 weeks of hyperglycemia. The animals were either untreated (n=7, D8 group) or treated with three different low doses of RSV: 1 mg/Kg, 2.5 mg/Kg and 5 mg/Kg/day (D8R_1mg, n=4; D8R_2.5mg, n=5; D8R_5mg, n=5). Age-matched normal rats were used as control group (C; n=7). Glucose blood levels and body weights were measured in 2-hour-fasting animals, before STZ or vehicle injection, two days after injection, and then weekly until sacrifice. Functional measurements were performed after 8 weeks of hyperglycemia, one hour after the last RSV injection. In a subgroup of animals, before sacrifice, urine (2 mL) and blood (4 mL) samples were also taken to determine urine and plasma levels of RSV and its glucuronide and sulfate conjugates. Plasma samples, separated by centrifugation at 1600 g for 15 minutes, and urine samples were then stored at -80°C .

Functional measurements:Hemodynamics

Rats were anaesthetized with ketamine chloride 40 mg/kg i.p. (Imalgene, Merial, Milano, Italy), plus medetomidine hydrochloride 0.15 mg/kg i.p. (Domitor, Pfizer Italia S.r.l., Latina, Italy). Invasive hemodynamic data were recorded with a microtip pressure transducer catheter (Millar SPC-320, Millar Instruments, Houston, TX, USA) connected to a recording system (Power Laboratory ML 845/4 channels, 2Biological Instruments, Besozzo, Italy). The following parameters were measured: 1) LV systolic pressure (LVSP), 2) LV end-diastolic pressure (LVEDP), 3) the peak rate of rise and decline of LV pressure (±dP/dt), taken as indexes of myocardial mechanical efficiency, 4) isovolumic contraction time (IVCT: duration of isovolumic contraction), 5) LV isovolumic relaxation time (LVRT), computed from –dP/dt to 5 mmHg above LVEDP [5], and 6) total cycle duration, contraction + relaxation (software package CHART B4.2).

Plasma and urine levels of RSV and RSV metabolites.

- Commercial Standards and Reagents

Trichloroacetic acid (TCA) was purchased from Sigma (St. Louis, MO, USA). All the liquid chromatography (LC) solvents were purchased from Carlo Erba Reagents (Milano, ITALY). RSV-3-*O*-glucuronide and RSV-3-*O*-sulphate were purchased from Bertin Pharma (Montigny le Bretonneux, FRANCE).

- Sample extraction

Proteins from plasma samples (800 µL) were precipitated with 160 µL 20% (v/v) TCA and then extracted with 800 µL cold methanol by vigorous vortexing for 10 min. The methanol extract obtained after centrifugation was dried under vacuum by rotary evaporation and then the residue dissolved in 200 µL of 50% (v/v) methanol acidified with 1% (v/v) formic acid for analysis.

- UHPLC-MS3 analysis

RSV metabolites were analyzed using an Accela UHPLC 1250 equipped with linear ion trap-mass spectrometer (LTQ XL, Thermo Fisher Scientific Inc., San Jose, CA, USA) fitted with a heated-electrospray ionization (H-ESI-II) probe (Thermo Fisher Scientific Inc., San Jose, CA, USA). Separations were performed using a Hypersil GOLD 1.9 µm Column (50x2.1 mm)(Thermo Fisher Scientific Inc.). The mobile phase, pumped at a flow rate of 0.3 mL/min, was a 7-min linear gradient of 3% to 48% acetonitrile in 0.1% aqueous formic acid. A constant 2% of methanol/water (98:2, v/v) was pumped during the gradient. The H-ESI interface worked in negative ionization mode. The capillary temperature was set to 275 °C, while the source heather temperature was 40 °C. The source voltage was 4 kV. The capillary voltage and tube lens were -50.00 and -127.71 V, respectively. The RSV metabolites in plasma were analyzed MS3 mode. The glucuronide forms of RSV, with a mass-to-charge ratio (m/z) at 403 were fragmented using a collision induced dissociation (CID) equal to 16 (arbitrary units), whereas sulfate conjugates (m/z 307) required a CID of 23 and then free RSV was further fragmented with a CID of 34. Pure helium gas was used for CID. RSV glucuronide isomers were quantified as RSV-3-*O*-glucuronide equivalents, while RSV sulfate isomers were quantified as RSV-3-*O*-sulfate equivalents.

Preliminary Results

At the beginning of the experimental protocol, glucose blood levels and body weight ranged respectively between 95-110 mg/dL and 354-436 g, in all groups of rats. After STZ injection, in diabetic animals blood glucose levels significantly increased as compared with normal values. However, hyperglycemia was slightly lower in D8R_2.5mg and D8R_5mg animals (440±11 mg/dL and 380±32 mg/dL, respectively) as compared with both untreated (520±5 mg/dL) and D8R_1mg rats (569±12.5 mg/dL). A 10% loss of body weight occurred during the first week of diabetes in all diabetic animals. Body weight remained essentially constant in D8R_2.5mg and D8R_5mg throughout the experimental protocol while a further progressive reduction (reaching 25% loss of the initial body weight, at the end of the experiments) was observed in untreated D8 and D8R_1mg groups.

As compared with C animals, marked changes in hemodynamic parameters were produced by 8 weeks of hyperglycemia in untreated animals (D8), indicating a global worsening of left ventricular hemodynamic efficiency. Specifically, D8 rats exhibited (i) a significant decrease in systolic LV pressure (range: 89-122 mmHg vs. 100-157 mmHg), (ii) a reduction in +dP/dt and –dP/dt (Figure S1 A-B), and (iii) a prolonged isovolumic contraction time (IVCT; Figure S1 C), relaxation time (LVRT; Figure S1 D) and total contraction-relaxation time (Figure S1 E). Although RSV administration induced an improvement of cardiac performance at all doses tested, the two higher doses (2.5 mg/Kg and 5mg/Kg) resulted in a total recovery of ventricular function while the lowest dose (1 mg/Kg) was less effective and did not prevent the relaxation impairment (Figure S1).

Plasma and urine levels of RSV active metabolites, measured on the three treated diabetic groups one hour after the last administration of the compound, are reported in Table S1. All the compounds were absent from urine and plasma samples of untreated diabetic and control rats. Despite the high intrinsic inter individual variability of one-hour absorption and metabolism of RSV and the low number of animals, we found a definite dose-dependent increase in plasmatic RSV-sulfate and urinary RSV-glucuronide concentrations. However, in plasma samples, the increase in the RSV-sulfate median value was sharp from 1 mg to 2.5 mg whereas it was less relevant when the fed dose went up to 5 mg (Table S1)

Conclusions

In summary, the results of the pilot study indicate that comparable positive effects on functional parameters recorded in diabetic animals can be obtained by chronic RSV administration, at the doses of 2.5 mg/Kg/day and 5mg/Kg/day. Both doses lead to a complete recovery of ventricular hemodynamic performance, while the lower dose (1 mg/Kg/day) was less effective. Dose dependent changes were detected in plasma (RSV-sulfate) and urinary (RSV-glucuronide) concentrations of RSV metabolites.

On the basis of these results, and by considering that poor information is so far available concerning the distribution of RSV and its active metabolites in cardiac tissue after long-term treatment with the compound, for the main study we selected the lowest dose capable of inducing functional recovery, i.e. 2.5 mg/Kg/day. It should be also outlined that this dose is equivalent to 27 mg/day for a 170cm/70 kg human subject (US Food and Drug Administration Center for Drug Evaluation and Research dose calculator). This dose is within the range easily attainable with virtually every good quality food integrator present on the market as well as well as lower than the average doses administered to human subjects [6-7].

**References**

1. Thirunavukkarasu M, Penumathsa SV, Koneru S, Juhasz B, Zhan L, et al. (2007) Resveratrol alleviates cardiac dysfunction in streptozotocin-induced diabetes: Role of nitric oxide, thioredoxin, and heme oxygenase. Free Radic Biol Med 43: 720-729.
2. Dudley J, Das S, Mukherjee S, Das DK (2009) Resveratrol, a unique phytoalexin present in red wine, delivers either survival signal or death signal to the ischemic myocardium depending on dose. Journal of Nutritional Biochemistry 20: 443–452.
3. Yar AS, Menevse S, Alp E, Helvacioglu F, Take G (2010) The effects of resveratrol on cyclooxygenase-1 and cyclooxygenase-2 mRNA and protein levels in diabetic rat kidneys. Mol Biol Rep 37: 2323-2331.
4. Lin JF, Lin SM, Chih CL, Nien MW, Su HH, et al. (2008) Resveratrol reduces infarct size and improves ventricular function after myocardial ischemia in rats. Life Sci 83: 313-317.
5. Myreng Y, Smiseth OA (1990) Assessment of left ventricular relaxation by Doppler echocardiography. Comparison of isovolumic relaxation time and transmitral flow velocities with time constant isovolumic relaxation. Circulation 81: 260–266.
6. Smoliga JM, Baur JA, Hausenblas HA (2011) Resveratrol and health--a comprehensive review of human clinical trials. Mol Nutr Food Res 55:1129-1141.
7. Magyar K, Halmosi R, Palfi A, Feher G, Czopf L, et a. (2012) Cardioprotection by resveratrol: A human clinical trial in patients with stable coronary artery disease. Clin Hemorheol Microcirc 50: 179-187.
